# A Lightweight IDS Based on Blockchain and Machine Learning for Detecting Physical Attacks in Wireless Sensor Networks

**DOI:** 10.3390/s26061961

**Published:** 2026-03-20

**Authors:** Maytham S. Jabor, Aqeel S. Azez, José Carlos Campelo, Alberto Bonastre

**Affiliations:** ITACA Institute, Universitat Politècnica de València (UPV), Camino de Vera s/n, 46022 Valencia, Spain; mayaz@upv.edu.es (M.S.J.);

**Keywords:** WSN, blockchain, IDS, physical attack, ANN, lightweight, intrusion detection

## Abstract

Wireless sensor networks (WSNs) are vulnerable to physical attacks in which adversaries gain partial or full control of sensor nodes, compromising the integrity of the network. Conventional security mechanisms impose excessive computational overhead and are not well suited to resource-constrained WSN devices. This paper proposes a lightweight, two-layer intrusion detection system (IDS) that integrates blockchain (BC) technology with machine learning for physical attack detection in WSNs. The first layer employs a lightweight BC protocol among cluster heads (CHs) and the base station (BS) to detect data integrity violations through hash-based consensus. The second layer applies an artificial neural network (ANN) at the base station to detect attacks that bypass blockchain verification, without imposing any processing load on sensor nodes. Simulation experiments on a 100-node WSN demonstrate that the combined system achieves 97.42% accuracy and 98.35% recall, outperforming five established classifiers and both standalone components. The system sustains detection rates above 99.98% under 30 simultaneous attackers and maintains reliable operation under packet loss conditions up to 10%.

## 1. Introduction

WSNs are composed of low-cost sensing nodes which cooperatively obtain and transmit data to a sink or BS [1]. WSNs have been successfully deployed in a wide range of application domains ranging from environmental monitoring to precision agriculture and health care systems [2,3]. The innate benefits of WSNs are the ability to provide a large spatial coverage, autonomous self-organization and intrinsic fault tolerance [4]. However, the scarce power resources and computational power of WSNs impose considerable limitations on their operation and often require significant human effort to maintain and manage these systems [5]. Security remains the most important issue in WSNs. Communication made over insecure wireless channels is vulnerable to a variety of threats such as eavesdropping, unauthorized data tampering and privacy degradation [6]. Attacks against WSNs are generally categorized as internal (insider) and external (outsider); attacks are harmful to the dependability and efficiency of network operations [7].

Cyber attacks on WSNs have far-reaching consequences across several dimensions. Eavesdropping intrusion allows for the illicit acquisition of data while compromising the confidentiality of data within a variety of application domains. Denial-of-service (DoS) attacks exhaust the energy reserve of the nodes by continuously requesting transmissions, effectively limiting the network longevity to sub-operational levels. Routing incursion—including sinkhole and wormhole variants—routes network traffic through malicious nodes that allow data interception and packet forwarding suppression on a per-packet basis. Violations of data integrity, where adversaries alter the measurements coming from the sensors, cause dishonest system reactions, especially when dealing with safety-critical operation contexts.

**Figure 1:**
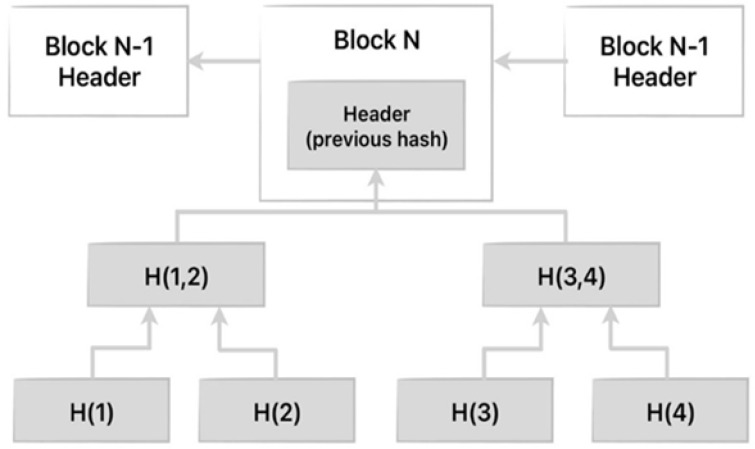
BC structure as used in the proposed IDS.

WSNs are further complicated by non-malicious threats that affect the quality of data as well as the reliability of the networks. Environmental factors such as temperature variations, rainfall, dust build-up and different humidity levels degrade the accuracy of both the sensors and the radio propagation characteristics. Hardware-induced noise and issues associated with the aging of components are factors that contribute to measurement drift over time. Electromagnetic interference from nearby electronic devices causes packets to be corrupted and causes communications to fail. Redundancy and duplicity in the coverage of the sensors adds processing overhead without corresponding informational gain. Such non-attack perturbations make intrusion detection difficult because anomalous sensor readings could be caused by benign environmental effects, not necessarily due to malicious intention, and hence increase the level of false positives in any IDS [8,9]. Accordingly, effective IDSs must have mechanisms to detect such benign anomalies in order to reduce false alarms while maintaining a high detection accuracy. Among these threats, there is a wide range of possible attacks to which WSNs can be vulnerable; a physical attack is one of the hardest to identify and has the potential to subvert most or all functionality in the system [10]. Physical attack detection and prevention remain an open issue in WSNs composed of resource-constrained nodes. Conventional security measures, such as heavy encryption algorithms and overhead-intensive IDSs, are not well suited for WSNs in terms of resource (battery, processing capability, memory, etc.) availability [11,12]. BC technology provides a promising alternative for securing distributed systems. This technology greatly promotes the security and privacy of information by addressing unauthorized access and mitigating the threat of cyber attacks effectively [13,14]. As illustrated in Figure 1, BC is simply an open ledger with a collection of blocks, and the data in one block is linked to its previous block using cryptography. Nevertheless, BC applications in WSNs should be wisely controlled in order to avoid undue resource consumption [15,16].

To solve the above problems, we propose a two-tier security framework designed for a resource-limited WSN topology. Furthermore, inspired by our prior work [1], where we demonstrated the benefits of employing a circuit tailored to minimize power consumption due to implementing BC in a WSN, an ANN architecture is proposed as an enhancement mechanism for the detection of attacks. In the two layers of the proposed approach, the first layer applies an optimized lightweight BC protocol that is suitable for a resource-constrained environment; while the secure encryption and validation of data generated by BC-connected nodes help preserve data integrity and identify malicious nodes. Indeed, as previously stated [1], our framework addresses the use of BC in WSN by limiting BC participation to select nodes, and offloading computationally intensive tasks to more capable nodes or the BS. In our proposal, the selected nodes that participate in BC are the CHs and BS. CHs are responsible for collecting data from nearby sensor nodes (SNs), aggregating it, and transmitting the processed data to a BS participating in the BC network. However, SNs are not connected to the BC. This has advantages from the point of view of resource consumption on the nodes, but may generate risky situations for attacks, which should be avoided by applying an additional mechanism to the BC. Moreover, BC is not immune to all possible attacks. It detects many types of attacks, both active and passive, but physical attacks that involve the attacker gaining full control of a CH will not be detected. This means the attacker has full access to the internal measurements of the compromised node, enabling them to read, modify, or replace sensor readings. For all these reasons, a second layer to identify errors not detected by the BC is needed. Figure 1 shows the proposed IDS’s BC structure. Each block contains sensor data aggregated by a CH, a timestamp, and a cryptographic hash of the previous block generated using chaotic hash functions. The linked hash chain is maintained among CHs and the BS. When the BS receives a new block, it verifies the hash against the expected chaotic sequence; any mismatch signals a Type A physical attack (incomplete node compromise). This tamper-evident structure forms the first detection layer of the proposed system.

The second layer utilizes machine learning (ML) techniques, specifically an ANN, to detect anomalies and unauthorized data manipulations from SNs that are not directly connected to the BC, and from a captured CH that attempts to corrupt the BC. By using ML at BS nodes, this proposal will mitigate the computational burden of SNs; it reduces resource consumption on hardware-limited nodes, and helps identify malicious nodes and forged data, which could compromise the network. Thus, the proposed two-level security structure provides an alternative suitable method to improve WSN security without consuming too many resources of the network. This hybrid model of lightweight BC for data integrity and ML algorithms for anomaly detection, and with new defensive strategies that support each other, forms an effective defense method that provides defense against both insider and outsider attacks. To minimize power consumption and computation complexity, the ANN works at the BS. Algorithms of ML and deep learning (DL) work efficiently with complex and non-linear data patterns. These techniques apply to IDSs, and attackers may want to design stealthy attacks [17]. The centralized processing at the BS allows real-time threat analysis and energy savings for all SNs. The generated ANN not only makes a significant performance gain over other lightweight schemes in WSNs, but also effectively balances security, reliability, and resource efficiency. It is one of the few algorithms that maintains a high accuracy and has a low computational demand, so that it can be used in a real-time application. ANNs are extremely powerful for discriminating complex and non-linear datasets, in addition to not having a rigid input condition, unlike several other algorithms, causing more flexibility in using them regarding various WSN applications [18,19]. The remainder of the paper is organized as follows: In Section 2, the related works are introduced, and previous research is discussed. A BC and an ANN trust mechanism designed for detecting malicious nodes in WSNs is introduced in Section 3. The experimental results and simulation evaluations of model execution analyses are described in Section 4. Last, Section 5 presents the conclusions of this work.

## 2. State of the Art

This section reviews the main research that has used BC and AI approaches to improve the security and performance of WSNs. In the research conducted by Almomani and Alenezi [20], random forest (RF) performed better than ANNs in terms of DoS alerts. The authors used different classifiers such as support vector machine (SVM), naive Bayes (NB), decision tree (DT), RF, J48, ANN, and k-nearest neighbors (KNN) with feature selection. However, the RF method is computationally expensive in contrast to other accurate approaches. Ismail et al. [21] described a hybrid security scheme based on BC and ML, applied to Internet of Things (IoT) sensor networks, where a BC prevention module and an ML detection module were defined. The above two stages involved identity verification and trusted management based on Light Smart contracts, as well as knowledge map learning using the Light GBM algorithm to detect malicious nodes. BC complexity and ML-induced network load remain persistent challenges in lightweight BC systems. Khan et al. [22] have addressed these challenges by integrating DL models with RMCV for detecting the malicious nodes. They applied a three-tier Low-Energy Adaptive Clustering Hierarchy (LEACH) model. The second layer utilized BC-based real-time message content validation (RMCV) as well as multiple DL models, e.g., ANN, convolutional neural network (CNN), long short-term memory (LSTM), and gated recurrent unit (GRU) to detect malicious nodes. The detection rate of the GRU model was up to 97%. This approach generated computational overhead at the CH level; other algorithms tested returned lower levels of accuracy. The Proof-of-Authority (PoA) consensus contributes to alleviating pressure on the network more effectively, compared to the proof-of-work (PoW) consensus. Revanesh and Sridhar [23] proposed a secure distributed routing protocol by using a deep convolutional neural network with salp swarm optimization (DCNN-SSO) and PoA BC. The model proposed secure routing in WSNs by combining BC and meta-heuristic DCNN-SSO. The method employed PoA-based BC for encryption and route authentication. The simulations outperformed other BC methods. The computational burden remained unaddressed. She et al. [24] presented the BC trust model (BTM) for malicious node detection in WSNs. Every node maintains a local BC to perform trust computation based on packet loss ratio, delay, and forward rate. Even though this BTM discovers and isolates malicious nodes, BC load and peripheral node attacks are not handled by it.

Almaiah [25] presented an intrusion detection model that authenticated sensor messages using BC technology for classifying the nodes. The simulation achieved 94.9% accuracy. The rotation increased the overhead in time and energy. The proactive defense model presented by Cho and Cho [26] shares the detected internal attacker list using BC-based trust mechanisms among sensor networks. The method found packet-dropping effects 59–67% more efficiently than conventional trust approaches. This did not totally solve the energy resource limitations of WSNs. Different from this work, the CBSigIDS architecture of Wenjuan et al. [27] integrates BC and distributed signature-based IDS in the IoT to form a signature data file. The CBSigIDS is now a good choice of signature exchange mechanism. One must overcome scalability, latency, and computational hurdles. The ABAS model of Mbarak et al. [28] detects and prevents jamming attacks in WSNs, against which a trust mechanism based on BC is proposed. The method uses a private BC to combat jamming but does not use a lightweight detector, and does not account for the resource-depleted condition (RDC).

Narayana, Midhunchakkaravarthy [29] proposed a BC-based method to detect malicious nodes in mobile ad hoc networks (MANETs). The model was able to better find the presence of malicious nodes at the expense of a higher computational cost and delay during checking for a block. Arifeen et al. [30] proposed a Sybil attack detection scheme for underwater WSN using BC in which the CH assesses the trust value of nodes and writes into the BC. As shown above, recent studies have suggested using AI, particularly ML and DL, for attack detection in WSNs; this has achieved promising results in distinguishing normal from malicious network behavior. BC integration provides immutability and resilience to attacks. However, significant challenges remain in improving WSN security using BC and AI techniques. The computational overhead of many AI algorithms is unsuitable for WSNs’ limited resources, and existing work does not adequately address physical attacks. Therefore, there is a need for lightweight mechanisms to detect logical and physical attacks without overburdening WSN resources. Table 1 compares the approaches according to AI algorithm, lightweight BC, attack type, detection method, and reported accuracy.

## 3. Proposed System

**Figure 2:**
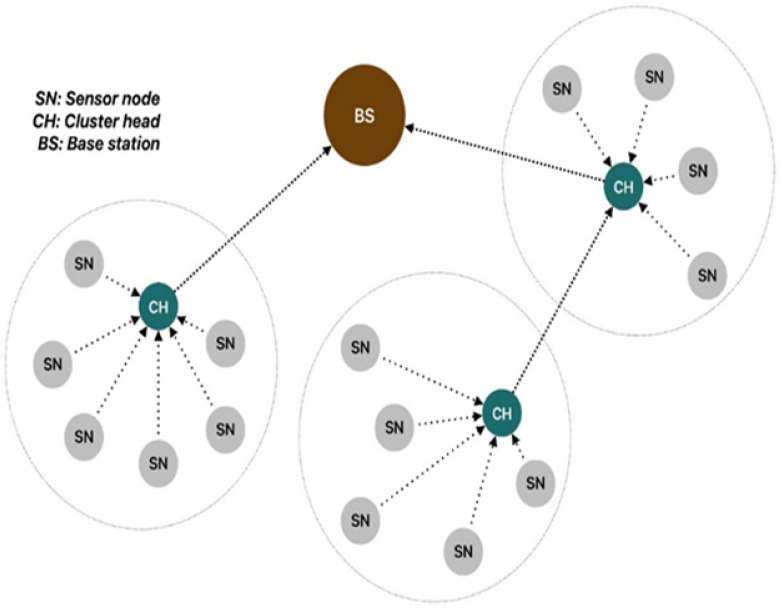
Clustering structure of the WSN.

This section presents our proposed two-tier security framework designed to enhance the security and efficiency of WSNs by integrating BC technology and artificial intelligence. The generic or common architecture of a WSN (Figure 2) is based on the grouping of nodes into clusters. In each cluster, one CH is responsible for aggregating and forwarding information to a BS or another CH. Consequently, the CH reduces the communication overhead by minimizing direct transmissions from individual nodes, thus conserving energy and extending the network’s lifespan. Our security proposal must operate on this common architecture and, at the same time, be generic enough to adapt to other modes of operation and diverse clustering protocols. While the LEACH protocol [31] is one of the possibilities due to its widespread use, and it is used in our experimentation, the framework is also applicable to WSNs without clustering protocols, including those with fixed CHs. BC is a distributed, tamper-evident ledger in which data are organized into a sequence of linked blocks. Each block contains a payload, a timestamp, and a cryptographic hash of the preceding block, forming a chain that makes retrospective data modification detectable without a central authority. In the WSN context, CHs act as block producers: each CH aggregates sensor readings from its member nodes, computes a hash using the chaotic hash parameters established during initialization, and submits the resulting block to the BS for consensus verification. If the hash does not match the expected value, the BS flags the block as tampered (Type A attack). The BC layer is lightweight for the network because hash computation is the only additional operation required at the CH, and all consensus logic runs at the BS. However, the BC layer has a fundamental limitation: an adversary with full node control can regenerate a valid hash after modifying the sensor data, bypassing hash-based detection entirely. This motivates the second detection layer.

The key idea of our two-tier security proposal is (i) to use BC among CHs and the BS for secure data management, while SN are not integrated into the BC in order to conserve their limited resources, (ii) to implement, in the BS, AI techniques based on ANN to enhance the attack detection capabilities of the BC system. Let us now examine in more detail the flow of messages sent, their integration into the BC, and the use of AI to detect attacks not identified by the BC technology. For more precise details on the implementation of BC, its use, operation, and the designed circuit, see [1].

### 3.1. Data Encryption and Transmission from SNs

**Figure 3:**
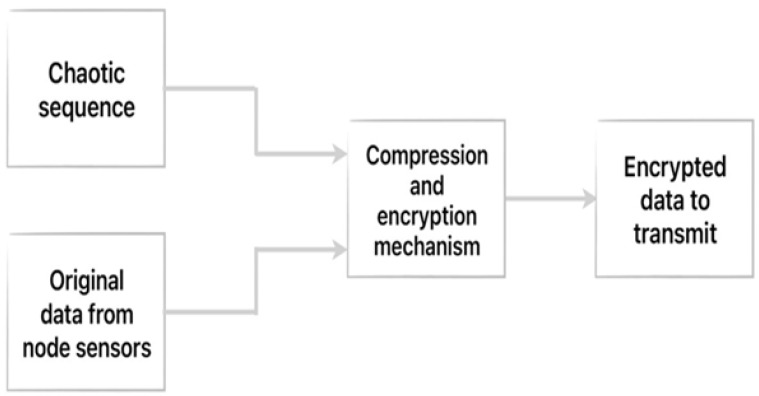
Sensing and encryption data.

As mentioned above, SNs are not connected to the BC in order to maintain their limited computational and energy resources. The price to pay for this decision, which minimizes resource consumption and extends the life of the WSN, is that we will have to ensure that attacks on the SNs are detected by some other method, as they will not be protected by the BC technology. This method must also be feasible from a consumption point of view (i.e., much better than including all nodes in the BC and the huge resource consumption that would entail). First, we will examine how SNs work. Although it is not mandatory, in earlier research [1] we proposed using encryption and compression methods for transmitting information from SN to CH. On the one hand, data compression will improve the efficiency of transmissions and minimize energy consumption, while on the other, encryption will make the information uninterpretable by attackers who want to access it [32,33]. However, these encryption and compression methods must be appropriate for use in WSNs, i.e., not generate excessive resource consumption. In our previously referenced work, we successfully proposed the use of compressed sensing (CS) techniques for these functions over other proposals. CS has many important features [1]: security analysis showed that it resists brute force attacks, is sensitive to secret keys, is robust against statistical attacks, and can use the parameters of the chaotic system as the secret key in the construction of the measurement matrix and the masking matrix. Moreover, the computational complexity of these operations is much lower than that of the current mainstream RSA encryption scheme. The unique properties of chaotic maps ensure that attackers cannot easily guess the encryption keys. In addition, the CS algorithm can satisfy all requirements of a hash function in an efficient and flexible manner. Accordingly, in our proposal, each node (SN and CH) is provisioned with a unique encryption key (parameters of the chaotic system) during startup, which is generated using chaotic maps; this ensures a high level of security due to their sensitivity to initial conditions and parameters [34,35]. These parameters are generated individually at each SN based on a deterministic approach using chaos theory. After reading data from the sensors (Figure 3), SN compresses (also encrypts) them using a chaotic logistic map.

Then, the result is transmitted to the CH. Parameters used in the chaotic map equation are transmitted along with the data [1,35,36], so that data can be deciphered when needed.

The encryption key K derived from the chaotic system is secured through multiple mechanisms. First, keys are generated locally at each SN using deterministic chaos theory with unique initial conditions (x0, μ); keys are never transmitted in plaintext across the network. The chaotic system exhibits a great sensitivity to initial conditions; a perturbation on the order of $10^{-15}$ in the initial parameter x0 results in the key sequence being quite different, thus making brute force enumeration unfeasible computationally. The continuity of parameter space inherent to chaotic maps (as opposed to the discrete key spaces common to conventional cryptography), makes an exhaustive search impracticable. Moreover, the inclusion of node-specific parameters ensures that the compromise of one node does not compromise the cryptographic keys of other nodes. If the enemy gains physical access to a node’s memory, which is designated as a Type B attack scenario in Section 3.4, the key contents stored in that memory are exposed, which is addressed by an ANN detection layer that identifies anomalous data patterns from compromised nodes regardless of their encryption status. A secondary security issue is introduced when parameters for a chaotic map are sent at the same time as compressed data is sent. An eavesdropper within radio range of the intra-cluster link could intercept these parameters. Various mitigating factors limit this risk.

The chaotic sequence evolves with each time step t, producing different parameters for each transmission; intercepting parameters for one message does not compromise future messages. The chaotic system sensitivity means that knowing current parameters does not enable the prediction of future parameters without the exact initial conditions stored in node memory. Two countermeasures can further address this vulnerability: (a) pre-sharing the chaotic parameters during node deployment rather than transmitting them at runtime, (b) encrypting the parameter transmission using a lightweight key exchange protocol. Even if parameters are captured and transmitted data is decrypted, the IDS maintains defense through the ANN analysis at the BS, which detects data manipulation regardless of encryption status. This limitation is acknowledged and addressed in future work. As outlined previously, this compression and encryption method allows efficient transmission with low energy demands, and attackers will have great difficulty, or a very low probability, of deciphering the content of the messages. However, physical data modification attacks (e.g., altering the sensor readings or modifying the memory locations where its readings reside, or even the message at the application level, before or after compression) will not be detected by the CH, as this will group the data from its SN to form a BC block, as we will see below. It should be noted that, in order not to overload the CHs and not to generate higher energy consumption, the data communicated by the SNs are not decrypted in CHs, but simply grouped together to form the new block, and the CS technique does not include mechanisms to ensure authenticity or integrity. As a CH receives apparently correct data, it will generate the block to be submitted for consensus in the BC. This situation calls for an additional mechanism to detect these attacks. Mathematically, the described operation can be represented as follows: Let CTE (chaos theory-based encryption) be represented by CTE($x_{0}$, μ, t), where $x_{0}$ is the initial condition, μ is the chaos control parameter, and t represents time. This function outputs the encryption key K.

Let CS be represented by CS(x, Φ, K), as a function that generates the encrypted data y, and y = Φx, where Φ is the sensing matrix. Then, the SN operation can be represented by the following equation:

(1) $$\mathrm{CS}(x,\Phi ,\mathrm{CTE}\left(x_{0},\mu ,t\right))$$

### 3.2. The BC and the ANN

Section 3.2 describes the two-layer operation of the proposed IDS: the BC layer, which runs among the CHs and the BS, and the ANN layer, which runs exclusively at the BS. Section 3.2.1, Section 3.2.2 and Section 3.2.3 cover block creation and consensus, DoS protection, and CH transition handling respectively. The ANN architecture and training procedure are detailed in Section 3.3.

#### 3.2.1. Blockchain Consensus and Block Creation

Upon receiving encrypted data from SNs, each CH aggregates the sensor readings from its member nodes and assembles them into a candidate block. Each block contains the aggregated payload, a timestamp, and a cryptographic hash of the previous block computed using chaotic hash functions (CHFs) [37,38]. The hash chain makes retrospective data modification detectable without a central authority. The BC consensus follows the first-come-first-served (FCFS) principle [1] rather than the traditional proof-of-stake (PoS) mechanism [39]. Each CH submits a bid to the BS proposing a new block. The BS maintains a schedule of bids in arrival order, selecting one CH per round to broadcast its block to all other CHs for validation. Upon successful validation, the BS distributes the approved block to all CHs, ensuring every node holds an up-to-date copy of the BC. The FCFS scheduling ensures equal participation among CHs and prevents any single node from dominating block creation.

#### 3.2.2. DoS Protection for the FCFS Mechanism

The FCFS consensus mechanism incorporates five protections against DoS attacks. (1) Authenticated bidding: the BS accepts bids only from CHs whose identity was verified during BC registration, blocking bids from unregistered nodes. (2) Rate limiting: each CH wins exactly one bidding slot per consensus round, preventing bid flooding by any single node. (3) Duplicate and misplaced bid rejection: bids that are redundant or temporally out of order are discarded by the BS before processing. (4) Limited attack surface: in a 100-node WSN with a 10% CH election probability, approximately 10 CHs are active per round, making large-scale spamming impractical. (5) Anomalous frequency detection: should a compromised CH attempt to flood the queue, the BS detects the unusually high bidding frequency and temporarily removes that CH from the schedule.

#### 3.2.3. CH Transition Handling

When a new CH is elected under the LEACH protocol, the outgoing CH transfers BC continuity by resubmitting recent block data to the incoming CH or by providing only the most recent blocks to preserve full BC integrity at the BS. The new CH joins the BC network without a mediator and participates directly in the consensus process, receiving a fresh copy of the BC from the BS. This mechanism ensures uninterrupted and secure operation through dynamic cluster structure changes without wasting network resources. Type A attacks are identified by the BC layer, where a node that has been compromised alters block data or the values of a hash before it is submitted. However, a basic limitation of the BC layer is the following: an attacker, who completely controls a CH, will be able to know the correct parameters of CS to be used in encryption and hashing, thus being able to produce a block that will seem legitimate but will have modified data. Such Type B attacks bypass BC verification entirely and require a separate detection mechanism, which is provided by the ANN layer described in Section 3.3. In this case, an ANN deployed at the BS is used. The ANN generates alerts from the data it receives from CHs and classifies each block as benign or malicious. The ANN was trained on both normal and abnormal data patterns, which allows it to detect forged data and compromised nodes. This second layer of defence is complementary to the BC security and provides protection against attacks that the BC scheme cannot detect. Since all computation occurs at the BS, no processing load is added to the SNs and CHs, and their resources remain conserved. The architecture and training procedure of the ANN are described in detail in Section 3.3.

**Figure 4:**
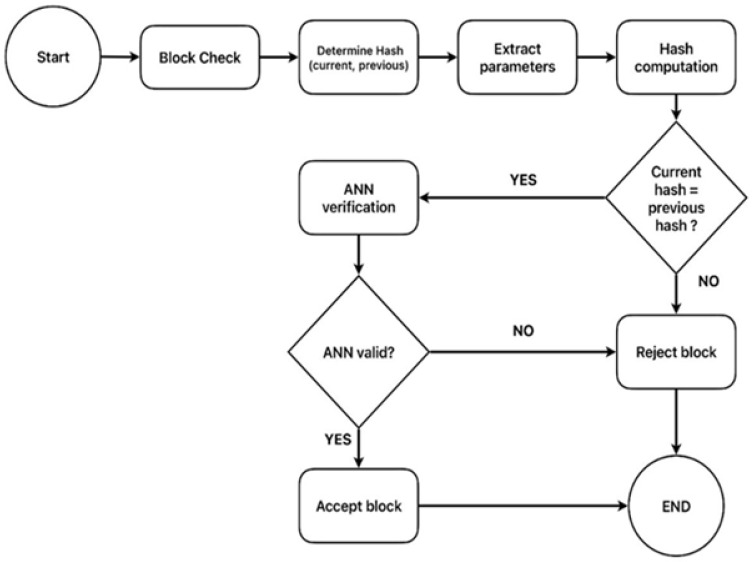
Mechanism for identifying malicious nodes at the base station (BS).

The process begins with hash verification. In the BS, as seen in Figure 4, when a new block is received and the ANN starts the operation, the hash value of each block generated by a chaos-based function is compared to the expected hash value. Any hash value mismatch identified is considered malicious, and processing stops. If the hash value is equal to the expected value, the ANN undergoes a more intensive analysis to identify data patterns that have been represented in the training data as per the principles of ML. The ANN also captures statistical properties such as mean, variance, and higher-order moments of the distribution of normal communication. Furthermore, the ANN captures the relationship between data points, making the ANN robust against advanced tampering techniques. To efficiently train the ANN, the payload data field and its timestamp are salient features. This timestamp is important for identifying anomalies in the communication pattern (e.g., the frequency of occurrence, delays between occurrences, time stamp differences between consecutive measurements or over time) which may indicate malicious behavior and, by learning about normal system behavior from non-malicious data, it gives information such as data value range/distribution and inconsistent transmission or variation, which can be used to infer a malicious value. Formalizing the proposed IDS, it can be represented as follows:

Let CHF(m, K) be a function that computes the hash code using chaos theory [1] of the data (m).

(2) $$\mathrm{CHF}\left(m,K\right)=\mathrm{CHF}\left(\mathrm{CS}\left(x,\Phi ,\mathrm{CTE}\left(x_{0},\mu ,t\right)\right),\mathrm{CTE}\left(x_{0},\mu ,t\right)\right)$$

Let BC(D, H) be a function that generates the candidate block, with data (D) and hash (H), to be included in the BC.

(3) $$\mathrm{BC}\left(D,H\right)=\mathrm{BC}\left(\mathrm{CS}\left(x,\Phi ,\mathrm{CTE}\left(x_{0},\mu ,t\right)\right),\mathrm{CHF}\left(m,K\right)\right)$$

Let ANN denote the network that analyses the candidate blocks to be included in the BC. Therefore, the lightweight IDS can be represented by

(4) $$\mathrm{IDS}=\mathrm{ANN}\left\{\mathrm{BC}\left(\mathrm{CS}\left(x_{i},\Phi_{i},\mathrm{CTE}\left(x_{0i},\mu_{i},t_{i}\right)\right),\;\mathrm{CHF}\left(\mathrm{CS}\left(x_{i},\Phi_{i},\mathrm{CTE}\left(x_{0n},\mu_{n},t_{n}\right)\right)\right)\right)\right\}$$

### 3.3. ANN Architecture and Training

An ANN is an approach to supervised learning which creates a mapping between input features and output classifications through a hierarchical structure of weighted transformations. In this architecture, the ANN is deployed in the BS and is trained using annotated block records of BC blocks so that it can distinguish between benign and malicious blocks received.

The ANN is selected over alternative classifiers because it handles the high-dimensional, non-linear feature space produced by the combined 576-feature block representation without requiring manual feature engineering. Centralizing ANN inference at the BS is a deliberate architectural decision: the BS operates from mains power and is not subject to the energy or memory constraints that govern sensor nodes and cluster heads. This design ensures that all machine learning overhead is absorbed by the BS, leaving the constrained portions of the network entirely unaffected. The ANN used at the BS has a diamond-shaped structure, which is designed to achieve a balance between the feature extraction capability and the computational efficiency. The input layer takes 576-dimensional feature vectors from the blocks in the BC, which include 512 data features (compressed readings from sensors), 32 hash features (current block hash), and 32 previous hash features. Four hidden layers with 128, 256, 512, and 256 hidden units and ReLU activation are used for processing these features. Each hidden layer is equipped with batch normalization (BN), which aids in training stability, dropout (dropout rate = 0.3), which helps prevent overfitting, and L1/L2 regularization (L1 = $1\times 10^{-5}$, L2 = $1\times 10^{-4})$ which helps regulate weights. The output layer uses the Softmax activation function with two classes, Benign and Malicious. Table 2 contains the detailed architecture. Training uses an Adam optimizer with a learning rate of 0.001, binary cross entropy as the loss function with a batch size of 64, and early stopping with a patience of fifteen epochs. The data used for training is based on a simulation of a WSN, in which each block in a BC is labeled “Attack” or “No Attack” based on the provenance of those blocks. In order to overcome the class imbalance between attack and benign traffic, Synthetic Minority Oversampling Technique (SMOTE) is used, which helps to balance the class distribution. Model performance is determined by means of five-fold stratified cross-validation to ensure a robust and unbiased estimation. The neural architecture has 374,914 trainable parameters.

### 3.4. Threat Model and Security Assumptions

The proposed IDS operates on the following security assumptions: (1) the BS is assumed to be completely trusted and is immune to compromise by an adversary; (2) more than 51% of CHs are assumed to be honest at any given time, which preserves the integrity of BC consensus; (3) adversaries are assumed to target individual SNs or CHs through physical access, but fail to compromise the BS simultaneously. Two categories of physical attacks are defined based on the level of access by the attacker. Type A Attack (Incomplete Compromise with Hash Inconsistency): In this case the attacker has physical access to a node but does not obtain the full set of CS parameters needed to correctly generate the hash. Consequently, although the attacker can manipulate sensor data or the behavior of nodes, any change that differs from the authentic configuration will break the process of chaotic hash computation, because the attacker does not have the precise initial conditions (x0, μ) to re-compute a valid hash. The resulting block generated in the compromised node has an inconsistent hash. BC consensus identifies such an attack by checking the hash against the expected chaotic sequence. This case describes a less capable adversary that is able to gain physical access without extracting all cryptographic material from the node memory. Type B Attack (Data Modification with Valid Hash): In this type of attack the attacker gains complete physical access to the node microcontroller, including all CS parameters and encryption keys. The attacker injects additive noise in the interval [−3.0,+3.0] into the sensor data and regenerates a cryptographically valid hash using the known parameters. Since the regenerated hash remains consistent with the modified data, this attack bypasses BC verification. Detection relies on the ANN, which identifies statistical deviations in the data features compared to the learned distribution of legitimate sensor readings. This attack requires full system knowledge and represents the most capable adversary in the threat model.

## 4. Results and Discussion

This section shows the results of the evaluation of the proposed IDS. The contribution of each of the two proposed security levels is obtained. To analyze the results, several metrics will be displayed: error detection rate, false positives and negatives obtained from the ANN, and network performance measures, namely throughput and packet delivery ratio (PDR).

### 4.1. Experimental Setup

The simulation is based on the LEACH protocol [31], which is a widely adopted WSN clustering scheme. In LEACH, nodes self-organize into clusters probabilistically each round: a node becomes a cluster head (CH) with a probability determined by its residual energy and a target CH fraction (*p* = 0.1 in this work). Each CH collects data packets from its member sensor nodes (SNs) via time division multiple access (TDMA) scheduling, aggregates them, and forwards the aggregated block to the base station (BS) in a single transmission. At the end of each round, the cluster structure is re-elected to distribute the energy burden across the network. This protocol is used in the experiments because it provides a realistic model of energy- limited WSN operation and is representative of cluster-based network architectures for which the proposed IDS is designed. The simulation was implemented in Python 3.10 using the TensorFlow 2.15 and Keras 2.15 libraries for ANN training and evaluation. All detection performance results in this section are reported using the following standard classification metrics. Let TP denote true positives (attack blocks correctly detected), TN true negatives (benign blocks correctly classified), FP false positives (benign blocks incorrectly flagged), and FN false negatives (attack blocks missed).

Accuracy = (TP + TN)/(TP + TN + FP + FN) is the proportion of all blocks classified correctly.

Precision = TP/(TP + FP) measures the fraction of flagged blocks that are genuine attacks.

Recall = TP/(TP + FN) measures the fraction of actual attack blocks detected; this is the primary security metric because undetected attacks (FN) are more costly than false alarms.

F1 = 2 × Precision × Recall/(Precision + Recall) is the harmonic mean of precision and recall, summarising detection quality when both FP and FN matter.

Inf. (ms) denotes the per-sample inference time in milliseconds, indicating the computational cost of classifying a single BC block in a real-time deployment. In our experiments, we simulated a WSN comprising 100 nodes in a 100 × 100 m area to generate a data set for training and testing the ANN and BC operation. The network simulation included normal SNs, CHs, BS, and malicious nodes. We used a generic application in which each node sends a message from a simulated sensor (which generates periodic transmissions of 50 bytes that are compressed and encrypted by CS) to its CH, following the operation described in Section 3. Performance evaluation uses five-fold stratified cross-validation with SMOTE oversampling for class balance.

Each BC block produces a 576-dimensional feature vector (512 data features + 32 hash features + 32 previous hash features). The two attack types defined in Section 3.4 are simulated with the parameters described below. Physical attacks can be classified based on the degree of control over the WSN node (SN or CH) the attacker gains. Type A (Incomplete Compromise): The attacker gains physical access to a node but does not obtain the CS parameters required for correct hash generation. Any modification to the node disrupts the chaotic hash computation, producing an inconsistent hash that the BC consensus detects. In the simulation, this is implemented by setting the chaotic map initial parameter to Xini = 0.1111, representing an attacker who lacks the correct cryptographic material. Type B (Full Compromise): The attacker gains complete access to the node microcontroller, including all CS parameters and encryption keys. The attacker modifies sensor data by injecting additive noise in the range [−3.0,+3.0] and regenerates a cryptographically valid hash. This attack bypasses BC verification, and detection relies on the ANN at the BS. The attacker was placed at different nodes (3, 40, 60, 70) across simulation sets to simulate spatial mobility and prevent detection methods from relying on node-specific anomalies.

### 4.2. ANN Classification Performance

Table 3 presents the ANN classification performance across five folds of stratified cross-validation. The model achieves a mean accuracy of 96.64% (±0.15%), precision of 88.68% (±0.77%), recall of 95.89% (±1.08%), and F1 score of 92.13% (±0.36%). The low standard deviations across folds confirm a stable and reproducible performance.

**Figure 5:**
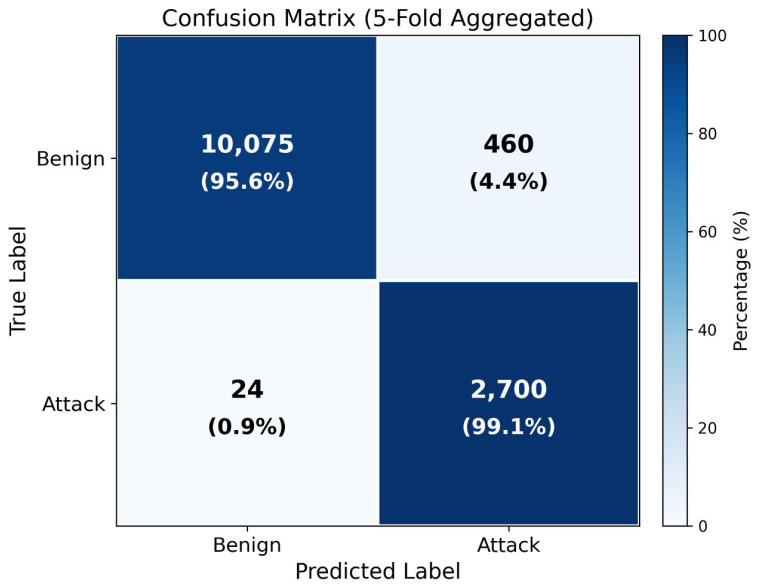
Confusion matrix from a representative train–test evaluation.

Figure 5 shows a confusion matrix from a representative single train–test evaluation. The matrix illustrates the classification behavior: the model correctly classifies 10,075 benign blocks and 2700 attack blocks, with 460 false positives and 24 false negatives. The definitive performance metrics are reported in Table 3 using five-fold stratified cross-validation (mean recall: 95.89% ± 1.08%, mean accuracy: 96.64% ± 0.15%), which provides a more robust estimate than any single split.

**Figure 6:**
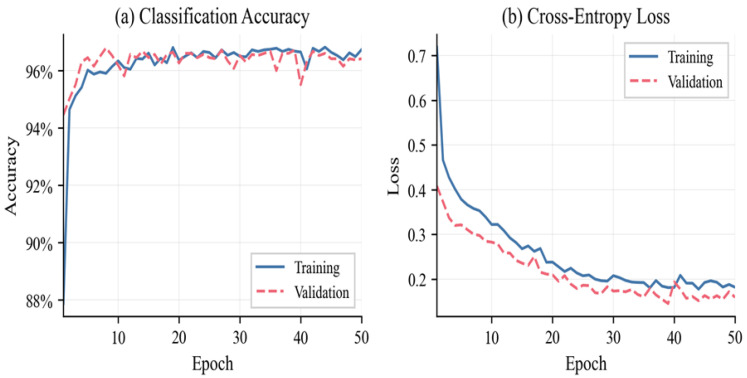
ANN training curves: loss (**a**) and accuracy (**b**).

Figure 6 illustrates the training and validation curve of the ANN. According to the graphs of the loss, convergence was obtained after thirty epochs, and the validation loss was similar to the training loss, thus indicating strong generalization and no overfitting. The figures of accuracy level off at about 96–97, which confirms the cross-validation.

### 4.3. Baseline Comparison

Table 4 compares the proposed ANN against five established classifiers using identical training data and five-fold stratified cross-validation. All models were trained on the same 576-feature BC block dataset. The proposed approach is the combined BC+ANN system; the standalone ANN row in Table 4 represents only one component of the full system. The combined system results (from the ablation study, Section 4.4) are included as the final row to allow direct comparison against all baselines. The combined system outperforms every baseline classifier simultaneously on accuracy (97.42% vs. RF 97.06%), recall (98.35% vs. SVM 100.00% and RF 98.27%), and F1 score (94.00% vs. SVM 93.24%).

**Figure 7:**
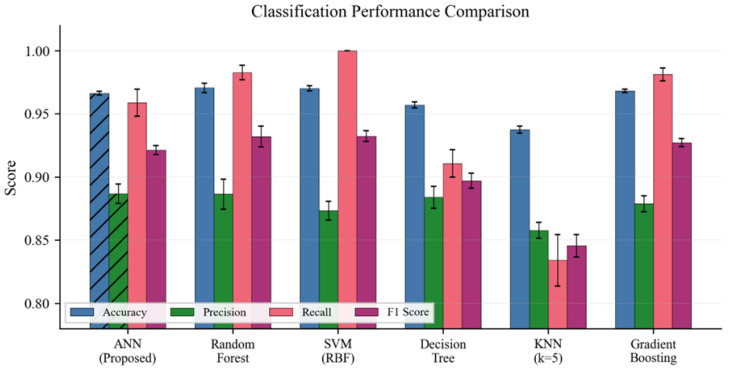
Classification performance comparison across baseline methods. Error bars represent the standard deviation across cross-validation folds.

The ANN achieves a 96.64% standalone accuracy, competitive with RF (97.06%) and SVM (97.02%). The accuracy gap (0.42% below RF) falls within the standard deviation of both models. The combined BC-ANN system (97.42% from the ablation study in Section 4.4) exceeds all standalone classifiers. Figure 7 illustrates the comparative performance across all metrics with error bars indicating standard deviation. The combined BC+ANN system row (final row of Table 4) demonstrates that the proposed approach achieves the highest accuracy (97.42%), the highest F1 score (94.00%), and a recall of 98.35% that surpasses all classifiers except SVM (100%). SVM achieves 100% recall but at the cost of a lower F1 score (93.24% vs. 94.00%), indicating a higher false positive rate. The combined system is the only method that simultaneously maximizes accuracy, F1, and competitive recall while maintaining a precision of 90.03%, which is the highest among all compared methods. Reference numbers for the baseline classifiers reflect the original algorithmic sources cited in the related work.

### 4.4. Ablation Study

Table 5 presents the ablation study results, comparing the three system configurations to quantify the contribution of each detection layer.

The BC alone achieves 88.34% accuracy but only 43.25% recall because it cannot detect Type B attacks where the attacker regenerates a valid hash after modifying data. Its precision of 100% indicates that every block it flags as malicious is confirmed as such (zero false positives), confirming that hash mismatch is a reliable indicator of Type A attacks. The ANN alone achieves 96.72% accuracy and 97.50% recall, demonstrating the strong data-driven detection of both attack types. The combined system achieves the highest performance (97.42% accuracy, 98.35% recall, 94.00% F1), confirming that BC and ANN provide complementary detection. The BC catches Type A attacks through hash verification, while the ANN detects Type B attacks through data pattern analysis. The combined recall (98.35%) exceeds ANN-only recall (97.50%) by 0.85%, quantifying the BC’s contribution to overall detection.

### 4.5. Computational Complexity Analysis

Table 6 provides quantitative metrics to evaluate the lightweight characteristics of the proposed ANN against all baseline classifiers.

The ANN uses 3.5× fewer parameters than SVM (1.33 M) and 32× fewer than KNN (12.1 M). It requires 3.6× fewer floating-point operations (FLOPs) per inference than SVM and 33× fewer than KNN. The model size (4,441 KB) is smaller than SVM (10,419 KB) and KNN (94,980 KB). While the ANN inference time (37.55 ms) is higher than tree-based methods, all ML computation occurs exclusively at the BS, which operates without energy or memory constraints. SNs and CHs are entirely free from ML overhead, making the system lightweight at the network level where resources are constrained.

To quantify the network-level lightweight property, two metrics are derived from the simulation data. First, communication overhead was derived as follows: the IDS relies on 64 hash features per block (32 hash + 32 previous hash), representing 2560 bytes per round across 10 CHs. This is 11.1% of the 23,040-byte block payload per round, with no additional message types required beyond standard blockchain operation. Second, energy overhead was derived as follows: simulation data across 70 fully operational rounds shows a network energy consumption of 0.5341 J/round (no attack) versus 0.5304 J/round (under Type B attack), a difference of less than 0.70% per round. Since all ANN inference (37.55 ms, 738,304 FLOPs) runs at the BS, sensor nodes and CHs bear no ML processing load. The IDS overhead on the constrained portions of the network is therefore negligible. Note that Table 6 compares model-level complexity metrics (parameters, FLOPs, inference time) for classifiers evaluated on the same BC block dataset. These metrics describe the model in isolation and are not directly comparable across deployment architectures. The lightweight characterisation of the proposed system applies at the network level, not the model level: all baseline classifiers in Table 6 are evaluated in a centralised test setting with no SN/CH deployment constraints, whereas the proposed ANN is deployed exclusively at the mains-powered BS by design. The sensor nodes and cluster heads bear zero ML overhead in the proposed system. This architectural distinction is the basis of the lightweight claim and is quantified by the energy and communication overhead figures reported above.

### 4.6. Multi-Attacker Scalability

**Figure 8:**
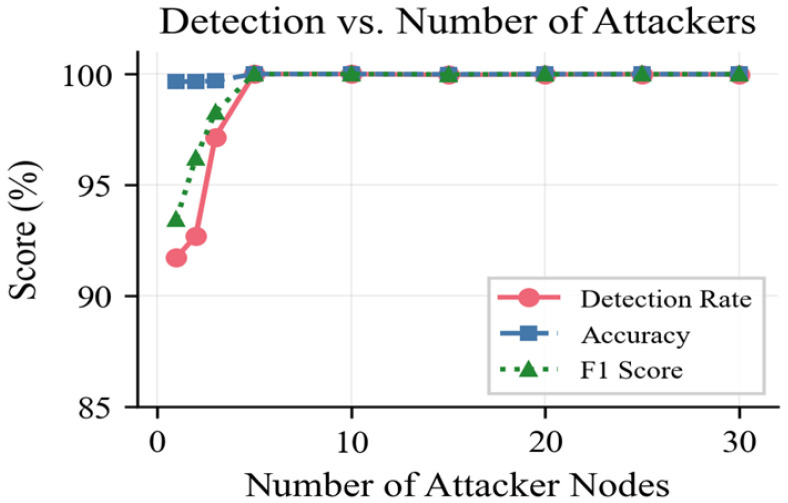
Detection rate versus number of simultaneous attackers.

Table 7 compares system performance with an increasing number of simultaneous attackers that vary from one to 30 (out of 100 nodes). The detection accuracy of the system is 91.72% when it confronts a single attacker and detection accuracy increased to 100% when the number of attackers increases up to five. The worse performance for detection when N = 1 is attributed to the fact that only a few attack samples can be used in ANN training, since if only one node is compromised, the attack blocks will represent only a small portion of the whole dataset, resulting in a lower statistical representation of attack patterns. Adding more attackers proportionally increases the amount of attack samples to the training data set so that the ANN has learned a more robust decision boundary. Detection accuracy is higher than 99.98% even in the case of thirty compromised nodes (30% of the network). The practical upper bound is controlled by the consensus requirement of the BC; provided there are more than 51% of the CHs remaining honest, the BC layer will function properly. The ANN also maintains its detection ability irrespective of the number of attackers because it analyses each block separately at the BS. Figure 8 shows the relationship between the detection rate and the number of attackers.

### 4.7. Network Performance Under Realistic Conditions

**Figure 9:**
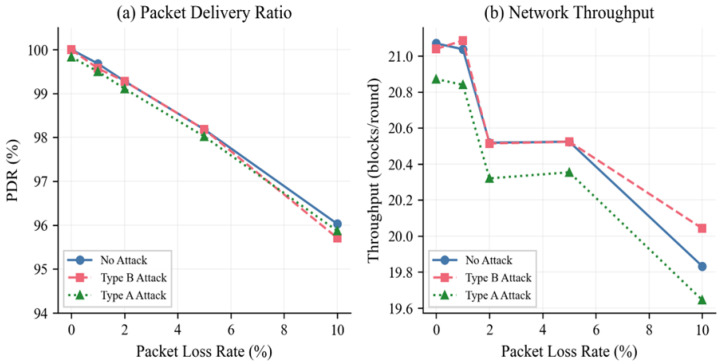
PDR (**a**) and throughput (**b**) under various packet loss rates.

Previous evaluation was based on ideal channel conditions (0% packet loss). To solve the problem of realism network impairments, in Table 8 we present PDR and throughput measurements at simulated packet loss rates of 0, 1, 2, 5, and 10%. When packet loss is zero, the PDR is 100 per cent in both the no-attack and Type B scenarios, thus making it an idealized simulation environment and separating the impact of the IDS from channel-layer imperfections. Type A attacks have a PDR of 99.83 per cent because of the BC consensus mechanism that rejects blocks with invalid hashes. Under more realistic conditions characterized by a packet loss rate of 10%, the PDR degrades gracefully to about 96%, showing the ability of the system to sustain reliable operation under adverse channel conditions. Throughput in blocks per round decreases in line with packet loss, from around 21 blocks per round with no packet loss to around 20 blocks per round when the packet loss rate is 10%. Type A attacks consistently result in lower throughputs when compared to the no-attack scenario, proving that the BC rejection of attacker blocks lowers the count of valid throughputs. The PDR and throughput degradation patterns are visualized in Figure 9.

**Figure 10:**
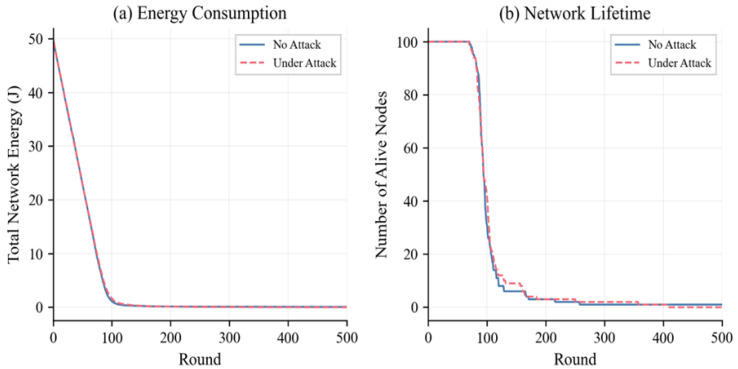
Energy depletion and alive nodes per round.

Figure 10 focuses on the loss of energy in the network and the survival of nodes from round to round of simulation. All 100 nodes are kept active until round 70 in the absence of attack and until round 69 with attack, which is in accordance with the expected LEACH energy model where nodes with the same initial energy (0.5 J) die simultaneously. The precipitous decline observed between rounds 71 and 100 is characteristic of LEACH clustering. The PDR is maintained at 100% (zero packet loss) throughout the simulation, as long as at least one node is still operational.

### 4.8. Feature Importance Analysis

Table 9 shows the feature group importance computed using RF feature importance scores, aggregated by feature group.

Data features carry 74.4% of the detection signal, confirming that the ANN primarily detects attacks through anomalies in the sensor data. Hash features contribute 25.1%, providing a complementary detection channel for Type A attacks. Previous hash features contribute minimally (0.5%), as they represent inherited chain state rather than current-block attack indicators. Table 10 presents the ANN feature ablation study, evaluating detection performance using different feature subsets.

**Figure 11:**
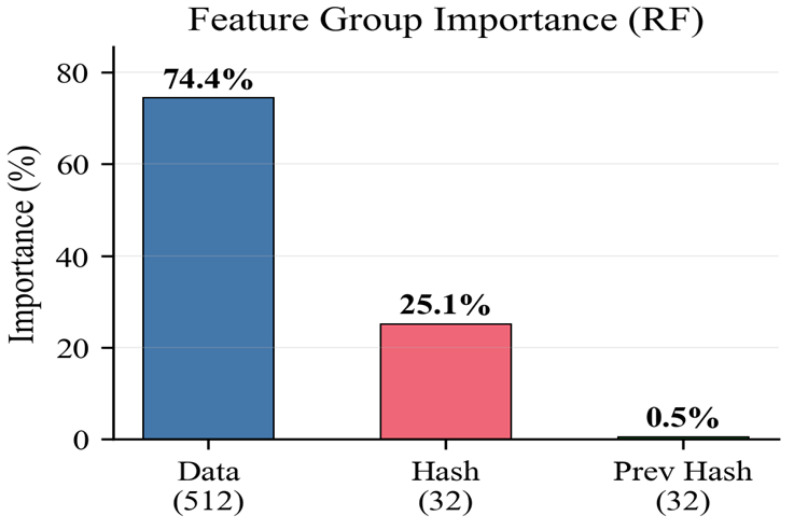
Feature group importance distribution.

Data-only features achieve the highest standalone accuracy (96.64%) and F1 (92.01%), confirming that sensor data analysis is the most effective detection mechanism Table 11. Hash-only features achieve 86.12% accuracy and 63.80% F1, showing limited detection capability for Type B attacks where hashes are regenerated. The combined feature set (96.18% accuracy) performs comparably to data-only features, indicating that the ANN primarily relies on data features. The combined system architecture addresses this by routing hash features through the BC layer (which processes them directly for Type A detection) while the ANN focuses on data features for Type B detection. Figure 11 illustrates the feature importance distribution.

The data-only model avoids this interference by excluding the ambiguous hash signal entirely. In the combined system, this is resolved architecturally: the BC layer handles hash-based Type A detection through consensus verification, while the ANN focuses on data patterns for Type B detection. The combined recall (98.35%) exceeds ANN-only recall (97.50%) by 0.85%, confirming that each component contributes to a distinct and complementary detection role.

## 5. Conclusions and Future Work

In this work, a bilayer IDS combining BC and ANNs to detect attacks in WSNs has been presented. Historically, centralized modalities performed malicious node detection in WSNs but without monitoring and verification of the original sensor data. Moreover, advanced physical attacks bypass classical detection techniques. However, BCs have now emerged as a promising decentralized technology for intrusion detection due to their consensus mechanism and tamper-evident logging capabilities. In the first layer of the proposed system, chaos theory and a simplified algorithm for hash generation were used to validate transactions, thereby simplifying block verification through the fragmentation produced by chaos theory. This approach successfully detects all the manipulated blocks when the attacker does not gain total access to the node; this demonstrates its high efficiency in detecting attacks and tampered data through the authentication method, using hash generation based on chaos theory. In the second layer, ANN algorithms were utilized to detect attacks in SNs that are not connected to the BC and in CH when an advanced attacker gains total access to secret parameters. In these situations, ANN infers the validity of the block before it is added to the BC, with high accuracy and precision. The combined system achieves 97.42% accuracy and 98.35% recall using five-fold stratified cross-validation, outperforming both standalone components (BC only: 43.25% recall; ANN only: 97.50% recall) and five baseline classifiers (RF, SVM, DT, KNN, GB). The system handles up to 30 simultaneous attackers (30% of the network) with detection rates above 99.98% and maintains PDR above 96% under 10% packet loss conditions. The BC layer helped to detect faulty nodes by verifying transactions and ensuring data integrity. Thus, the system could defend itself against attacks by distinguishing which nodes might have turned rogue, and implementing the corresponding countermeasures. The system maintained data integrity, as IDS monitored the data packets from the source to the destination points, and did not pass unauthorized forms or content. Several limitations of the present investigation should be acknowledged. The WSN simulation was based on an idealized radio propagation model that does not include multipath fading or shadowing phenomena. Node placement followed a uniform random distribution instead of reflecting application-specific deployment patterns. Although the LEACH protocol provides a well-analyzed benchmark, in realistic deployments, heterogeneous or energy-aware clustering protocols could be used. The attack model only included two types of physical attacks; in the real-world, adversaries may use other attack types such as replay attacks or coordinated attacks with multiple vectors. The training data for the ANN was obtained from the simulation; the transfer of the model to data from actual sensors may require fine-tuning to account for hardware-specific noise characteristics and deployment-specific data distributions. Future work will involve testing the framework in a physical hardware setup to better assess latency, response times, and resilience under realistic network conditions, including variable transmission delays and processing loads. Additional attack types including selective forwarding, replay attacks, and coordinated multi-vector assaults will be evaluated. Federated learning approaches will be explored to enable distributed ANN training across multiple BSs in large-scale deployments. The integration of integrity checking mechanisms in CS, such as error checking codes and verification hash codes, will be evaluated to reduce dependence on the ANN layer for Type B attack detection. Detecting whether the compressed information has been altered when decrypting or reconstructing the data remains a challenge in CS, since the technique does not include authenticity or integrity mechanisms. This evaluation will generate an overhead in resource consumption, which will need to be weighed and compared with the findings of this study. In addition, the experiments will be expanded to include multiple environments and different standards.

## Figures and Tables

**Table 1:** Comparison of the proposed survey studies.

Ref.	AI Algorithm	Lightweight BC	Attack Type	Detection Method	Accuracy	Dataset
[20]	RF, ANN	-	DoS	ML classification	-	KDD
[21]	LightGBM	Lightweight	Malicious node	Hybrid	-	Simulated
[22]	ANN, CNN, LSTM, GRU	-	Malicious node	DL classification	97%	Simulated
[23]	DCNN-SSO	-	Routing	DL + BC	-	Simulated
[24]	-	-	Malicious node	Trust-based	-	-
[25]	-	-	Malicious node	BC authentication	94.9%	-
[26]	-	-	Packet drop	Trust + BC	-	Simulated
[27]	-	-	Various	Signature IDS	-	-
[28]	-	-	Jamming	Trust + BC	-	Simulated
[29]	-	-	Malicious node	BC validation	-	-
[30]	-	-	Sybil	Trust + BC	-	Simulated
[Proposed]	ANN (Diamond 576-128-256-512-256-2)	Yes	Physical (Type A + B)	BC + ANN	97.42%	Simulated

**Table 2:** ANN architecture details.

Layer	Units	Activation	Regularization	Notes
Input	576	-	-	512 data + 32 hash + 32 prev_hash
Hidden 1	128	ReLU	L1L2 + BN + Dropout (0.3)	Compression layer
Hidden 2	256	ReLU	L1L2 + BN + Dropout (0.3)	Expansion
Hidden 3	512	ReLU	L1L2 + BN + Dropout (0.3)	Peak capacity
Hidden 4	256	ReLU	L1L2 + BN + Dropout (0.3)	Compression
Output	2	Softmax	-	Benign/Malicious

**Table 3:** ANN per-fold cross-validation results.

Fold	Accuracy (%)	Precision (%)	Recall (%)	F1 Score (%)
1	96.46	89.08	94.31	91.62
2	96.91	89.44	96.33	92.76
3	96.57	87.21	97.61	92.12
4	96.64	88.91	95.60	92.13
5	96.61	88.74	95.59	92.04
Mean	96.64 ± 0.15	88.68 ± 0.77	95.89 ± 1.08	92.13 ± 0.36

**Table 4:** Classification performance comparison with baseline methods.

Method	Accuracy (%)	Precision (%)	Recall (%)	F1 (%)	Inf. (ms)
ANN (standalone)	96.64±0.15	88.68±0.77	95.89±1.08	92.13±0.36	37.55
RF	97.06±0.37	88.65±1.19	98.27±0.58	93.21±0.83	0.013
SVM (RBF)	97.02±0.20	87.34±0.73	100.00±0.00	93.24±0.42	0.530
DT	95.71±0.24	88.40±0.86	91.08±1.09	89.71±0.58	0.001
KNN (k = 5)	93.75±0.28	85.78±0.62	83.41±2.04	84.56±0.89	0.475
GB	96.83±0.14	87.88±0.63	98.13±0.51	92.72±0.31	0.003
*BC + ANN (proposed)	97.42	90.03	98.35	94.00	37.55

*BC + ANN inference time reflects ANN inference only; blockchain hash verification adds negligible overhead. Method abbreviations: RF = Random Forest [20]; SVM = Support Vector Machine [40]; DT = Decision Tree [41]; KNN = K-Nearest Neighbors [42]; GB = Gradient Boosting [43].

**Table 5:** Ablation study: contribution of each detection layer.

Method	Accuracy (%)	Precision (%)	Recall (%)	F1 (%)
BC Only	88.34	100.00	43.25	60.38
ANN Only	96.72	87.87	97.50	92.43
Combined (Proposed)	97.42	90.03	98.35	94.00

**Table 6:** Computational complexity comparison.

Model	Parameters	Size (KB)	FLOPs	Inf. (ms)	Mem. (KB)
ANN (Proposed)	374,914	4440.8	738,304	37.55	120.2
RF	293,464	5763.8	6178	2.25	13.3
SVM (RBF)	1,329,985	10,418.7	2,655,360	0.88	6.9
DT	1820	36.7	58	0.05	6.6
KNN (k = 5)	12,136,320	94,980.3	24,272,640	12.36	172.8
GB	5984	133.2	600	0.13	7.0

**Table 7:** IDS network-level overhead summary.

Metric	Value	Notes
ML overhead on SNs/CHs	Zero	All ANN inference at BS (mains-powered)
Communication overhead	11.1% of block payload	2560 bytes/round across 10 CHs; no extra message types
Energy overhead	<0.70% per round	0.5341 J/round (no attack) vs. 0.5304 J/round (Type B)

**Table 8:** Detection performance versus number of simultaneous attackers.

N	Blocks	Det. Rate (%)	Acc. (%)	Prec. (%)	Recall (%)	F1 (%)
1	290	91.72	99.66	95.64	91.73	93.45
2	521	92.71	99.66	100.00	92.71	96.21
3	1054	97.15	99.69	99.42	97.15	98.27
5	1736	100.00	100.00	100.00	100.00	100.00
10	3154	100.00	100.00	100.00	100.00	100.00
15	5232	99.96	99.99	100.00	99.96	99.98
20	6030	99.98	99.99	100.00	99.98	99.99
25	8258	99.99	99.99	100.00	99.99	99.99
30	10,843	99.98	99.99	100.00	99.98	99.99

**Table 9:** PDR and throughput under various packet loss conditions.

Loss	PDR No Atk	PDR Type B	PDR Type A	Thr. No Atk	Thr. Type B	Thr. Type A
0%	100.00%	100.00%	99.83%	21.07	21.04	20.87
1%	99.68%	99.57%	99.50%	21.04	21.09	20.84
2%	99.28%	99.28%	99.11%	20.52	20.51	20.32
5%	98.18%	98.18%	98.02%	20.52	20.52	20.35
10%	96.03%	95.71%	95.87%	19.83	20.04	19.64

**Table 10:** Feature group importance (RF).

Feature Group	Number of Features	Importance (%)
Data Features	512	74.4
Hash Features	32	25.1
Previous Hash Features	32	0.5

**Table 11:** Feature ablation: ANN performance with different feature subsets.

Feature Set	Accuracy (%)	Precision (%)	Recall (%)	F1 (%)
Data Only (512)	96.64 ± 0.33	90.43 ± 4.63	94.05 ± 3.91	92.01 ± 0.45
Hash Only (64)	86.12 ± 0.38	68.77 ± 1.42	59.51 ± 0.32	63.80 ± 0.69
All Features (576)	96.18 ± 0.24	89.25 ± 1.40	92.62 ± 3.15	90.85 ± 0.82

## Data Availability

The data presented in this study are available on request from the corresponding author.

## Abbreviations

The following abbreviations are used in this manuscript:

WSN	Wireless Sensor Network
CH	Cluster Head
BC	Blockchain
ANN	Artificial Neural Network
DL	Deep Learning
CTE	Chaos Theory Encryption
LEACH	Low-Energy Adaptive Clustering Hierarchy
PDR	Packet Delivery Ratio
SVM	Support Vector Machine
KNN	K-Nearest Neighbors
FLOPs	Floating Point Operations
ReLU	Rectified Linear Unit
BS	Base Station
SN	Sensor Node
IDS	Intrusion Detection System
ML	Machine Learning
CS	Compressed Sensing
CHF	Chaos Hash Function
FCFS	First Come First Served
RF	Random Forest
DT	Decision Tree
GB	Gradient Boosting
SMOTE	Synthetic Minority Oversampling
PoS	Proof of Stake

## References

[1] Jabor M.S., Azez A.S., Campelo J.C., Bonastre Pina A. (2023). New approach to improve power consumption associated with blockchain in WSNs. PLoS ONE.

[2] Khan A.U., Javaid N., Othman J.B. (2021). A Secure Authentication and Data Sharing Scheme Based on Blockchain. Proceedings of the 2021 IEEE Symposium on Computers and Communications (ISCC).

[3] Kipongo J., Swart T.G., Esenogho E. (2023). Design and Implementation of Intrusion Detection Systems Using RPL and AODV Protocols-Based Wireless Sensor Networks. Int. J. Electron. Telecommun..

[4] Kim T.H., Goyat R., Rai M.K., Kumar G., Buchanan W.J., Saha R., Thomas R. (2019). A Novel Trust Evaluation Process for Secure Localization Using a Decentralized Blockchain in Wireless Sensor Networks. IEEE Access.

[5] Martey A.S., Esenogho E. (2022). Improved Cluster to Normal Ratio Protocol for Increasing the Lifetime of Wireless Sensor Networks. Indones. J. Electr. Eng. Comput. Sci..

[6] Mo J., Hu Z., Shen W. (2022). A Provably Secure Three-Factor Authentication Protocol Based on Chebyshev Chaotic Mapping for Wireless Sensor Network. IEEE Access.

[7] Javaid N. (2022). A Secure and Efficient Trust Model for Wireless Sensor IoTs Using Blockchain. IEEE Access.

[8] Kenyeres M., Kenyeres J., Hassankhani Dolatabadi S. (2025). Distributed Consensus Gossip-Based Data Fusion for Suppressing Incorrect Sensor Readings in Wireless Sensor Networks. J. Low Power Electron. Appl..

[9] Heidari A., Amiri Z., Jabraeil Jamali M.A., Jafari N. (2025). Assessment of Reliability and Availability of Wireless Sensor Networks in Industrial Applications by Considering Permanent Faults. Concurr. Comput. Pract. Exp..

[10] Keerthika M., Shanmugapriya D. (2021). Wireless Sensor Networks: Active and Passive attacks - Vulnerabilities and Countermeasures. Glob. Transit. Proc..

[11] Su G., Zhang B. (2025). Synergized Security Framework: Revolutionizing Wireless Sensor Networks through Comparative Methodological Analysis. Sci. Rep..

[12] Aminanto M.E., Wicaksono R.S.H., Aminanto A.E., Tanuwidjaja H.C., Yola L., Kim K. (2022). Multi-Class Intrusion Detection Using Two-Channel Color Mapping in IEEE 802.11 Wireless Network. IEEE Access.

[13] Mansour R.F. (2022). Blockchain Assisted Clustering with Intrusion Detection System for Industrial Environment. Expert Syst. Appl..

[14] Valadares D.C.G., Perkusich A., Martins A.F., Alshawki M.B., Seline C. (2023). Privacy-Preserving Blockchain Technologies. Sensors.

[15] Liu G., Zhao H., Fan F., Liu G., Xu Q., Nazir S. (2022). An Enhanced Intrusion Detection Model Based on Improved kNN in WSNs. Sensors.

[16] Guo F., Yu F., Zhang H., Ji H., Liu M., Leung V. (2020). Adaptive Resource Allocation in Future Wireless Networks with Blockchain and Mobile Edge Computing. IEEE Trans. Wirel. Commun..

[17] Kumari M., Pramanick N., Agarwal M., Esenogho E. (2025). An Optimized IDS Framework for Big Data Environments: Integrating Gravitational Search and SMOTE-IPF Data Balancing for High-Accuracy IDS. SN Comput. Sci..

[18] Ahmad R., Wazirali R., Abu-Ain T. (2022). Machine Learning for Wireless Sensor Networks Security: An Overview of Challenges and Issues. Sensors.

[19] Pandey V.K., Prakash S., Ojha G., Yang T., Rathore R.S. (2025). An Efficient Data Driven Model for WSN using Artificial Neural Network. Procedia Comput. Sci..

[20] Almomani I., Alenezi M. (2018). Efficient Denial of Service Attacks Detection in Wireless Sensor Networks. J. Inf. Sci. Eng..

[21] Ismail S., Nouman M., Dawoud D.W., Reza H. (2024). Towards a Lightweight Security Framework Using Blockchain and Machine Learning. Blockchain Res. Appl..

[22] Khan Z.A., Amjad S., Ahmed F., Almasoud A.M., Imran M., Javaid N. (2023). A Blockchain-Based Deep-Learning-Driven Architecture for Quality Routing in Wireless Sensor Networks. IEEE Access.

[23] Revanesh M., Sridhar V. (2021). A Trusted Distributed Routing Scheme for Wireless Sensor Networks Using Blockchain and Meta-Heuristics-Based Deep Learning Technique. Trans. Emerg. Telecommun. Technol..

[24] She W., Liu Q., Tian Z., Chen J., Wang B., Liu W. (2019). Blockchain Trust Model for Malicious Node Detection in Wireless Sensor Networks. IEEE Access.

[25] Almaiah M.A. (2021). A New Scheme for Detecting Malicious Attacks in Wireless Sensor Networks Based on Blockchain Technology. Artificial Intelligence and Blockchain for Future Cybersecurity Applications.

[26] Cho K., Cho Y. (2020). HyperLedger Fabric-Based Proactive Defense against Inside Attackers in the WSN with Trust Mechanism. Electronics.

[27] Li W., Tug S., Meng W., Wang Y. (2019). Designing Collaborative Blockchained Signature-Based Intrusion Detection in IoT Environments. Future Gener. Comput. Syst..

[28] Mbarek B., Ge M., Pitner T. (2022). An Adaptive Anti-Jamming System in HyperLedger-Based Wireless Sensor Networks. Wirel. Netw..

[29] Narayana V.L., Midhunchakkaravarthy D. (2020). A Time Interval-Based Blockchain Model for Detection of Malicious Nodes in MANET Using Network Block Monitoring Node. Proceedings of the 2020 Second International Conference on Inventive Research in Computing Applications (ICIRCA).

[30] Arifeen M.M., Al Mamun A., Ahmed T., Kaiser M.S., Mahmud M., Kaiser M.S., Bandyopadhyay A., Mahmud M., Ray K. (2021). A Blockchain-Based Scheme for Sybil Attack Detection in Underwater Wireless Sensor Networks. Proceedings of the International Conference on Trends in Computational and Cognitive Engineering: Proceedings of TCCE 2020.

[31] Farooq M.O., Dogar A.R., Shah G.A. (2010). MR-LEACH: Multi-Hop Routing with Low Energy Adaptive Clustering Hierarchy. Proceedings of the 4th International Conference on Sensor Technologies and Applications.

[32] Zhang P., Wang S., Guo K., Wang J. (2018). A Secure Data Collection Scheme Based on Compressive Sensing in Wireless Sensor Networks. Ad Hoc Netw..

[33] Zhang Y., Xiang Y., Zhang L.Y., Rong Y., Guo S. (2019). Secure Wireless Communications Based on Compressive Sensing: A Survey. IEEE Commun. Surv. Tutor..

[34] Ponuma R., Amutha R. (2019). Encryption of Image Data Using Compressive Sensing and Chaotic System. Multimed. Tools Appl..

[35] Nesa N., Ghosh T., Banerjee I. (2019). Design of a Chaos-Based Encryption Scheme for Sensor Data Using a Novel Logarithmic Chaotic Map. J. Inf. Secur. Appl..

[36] Alwan N.A.S., Hussain Z.M. (2019). Compressive Sensing with Chaotic Sequences: An Application to Localization in Wireless Sensor Networks. Wirel. Pers. Commun..

[37] Amin M., Faragallah O.S., Abd El-Latif A.A. (2009). Chaos-Based Hash Function (CBHF) for Cryptographic Applications. Chaos Solitons Fractals.

[38] Wu Q. (2015). A Chaos-Based Hash Function. Proceedings of the 2015 International Conference on Cyber-Enabled Distributed Computing and Knowledge Discovery (CyberC).

[39] Cho H. (2018). ASIC-Resistance of Multi-Hash Proof-of-Work Mechanisms for Blockchain Consensus Protocols. IEEE Access.

[40] Cortes C., Vapnik V. (1995). Support-vector networks. Mach. Learn..

[41] Quinlan J.R. (1986). Induction of decision trees. Mach. Learn..

[42] Cover T., Hart P. (1967). Nearest Neighbor Pattern Classification. IEEE Trans. Inf. Theory.

[43] Friedman J.H. (2001). Greedy Function Approximation: A Gradient Boosting Machine. Ann. Stat..

